# Examining the equivalence between imagery and execution within the spatial domain – Does motor imagery account for signal-dependent noise?

**DOI:** 10.1007/s00221-020-05939-z

**Published:** 2020-10-21

**Authors:** James W. Roberts, Greg Wood, Caroline J. Wakefield

**Affiliations:** 1 Psychology, Action and Learning of Movement (PALM) Laboratory, School of Health Sciences, Liverpool Hope University, Hope Park, Liverpool, L16 9JD UK; 2 Department of Sport and Exercise Science, Faculty of Science and Engineering, Research Centre for Musculoskeletal Science and Sports Medicine, Manchester Metropolitan University, Manchester, UK; 3 Present Address: Brain and Behaviour Laboratory, Research Institute of Sport and Exercise Sciences (RISES), Liverpool John Moores University, Byrom Street, Tom Reilly Building, Liverpool, L3 5AF UK

**Keywords:** Functional equivalence, Aiming, Motor noise, Effective target width

## Abstract

Motor imagery is suggested to be functionally equivalent to physical execution as they each utilise a common neural representation. The present study examined whether motor imagery correspondingly reflects the spatial characteristics of physically executed movements, including the signal-dependent noise that typically manifests in more variable end locations (as indicated by effective target width; W_e_). Participants executed or imagined a single, upper-limb target-directed aim in the horizontal medio-lateral direction. The start and end of the imagined movements were indexed by the lifting and lowering of the limb over the home position, respectively. Following each imagined movement, participants had to additionally estimate their imagined end location relative to the target. All the movements had to be completed at a pre-specified criterion time (400 ms, 600 ms, 800 ms). The results indicated that the W_e_ increased following a decrease in movement time for execution, but not imagery. Moreover, the total error of imagined movements was greater than the actual error of executed movements. While motor imagery may comprise a neural representation that also contributes to the execution of movements, it is unable to closely reflect the random sources of variability. This limitation of motor imagery may be attributed to the comparatively limited efferent motor signals.

## Introduction

For many years, there has been great interest surrounding motor imagery, where bodily movements are mentally simulated without physically executing the movement itself. This interest has no doubt manifested from the benefits that are served by imagery including the learning (Romano-Smith et al. 2018, 2019; Vogt, 1995) and re-learning (Crosbie et al. 2004; Dijkerman et al. 2004; Braun et al. 2013) of motor skills. Perhaps the most common or accepted theoretical framework surrounding motor imagery involves the concept of functional equivalence, which states that the neural representation that is responsible for the execution of movement is the same as that for the imagery of movement (Jeannerod 1994, 1999). This concept has been strongly supported by evidence of imagery and execution correspondingly activating the fronto-parietal neural regions (Filimon et al. 2007; Hétu et al. 2013). Moreover, there is evidence that imagery can modulate the corticospinal excitability (e.g., opponense pollicis during imagined forearm extension/flexion) that is generated by transcranial magnetic stimulation at the primary motor cortex (M1) (Fadiga et al. 1999).

Numerous studies have provided supporting evidence at the behavioural level by demonstrating that the task constraints influencing executed movement times may correspondingly influence imagined movement times (Decety and Jeannerod 1995; Glover and Dixon 2013; Gueugneau et al. 2017; Papaxanthis et al. 2002; Radulescu et al. 2010; Roberts et al. 2019; Rozand et al. 2015; Sirigu et al. 1995; Sirigu et al. 1996; Slifkin 2008; for a review, see Guillot and Collet 2005). Specifically, the greater the task difficulty, then the longer it takes to execute and imagine the movements that are required to complete the task. This principle is heavily adapted from the inherent trade-off between movement speed and accuracy (Woodworth 1899), which can be more formally expressed by a logarithmic function known otherwise as Fitts’ Law (Fitts 1954; Fitts and Peterson 1964). This law of human movement is able to predict movement time using the formula: *MT* = *a* + *b*(*ID*), where *a* and *b* represent the intercept and slope coefficient, respectively. Meanwhile, the *ID* represents the index of difficulty, which can be defined as: log_2_(2*A*/*W*), where *A* and *W* represent the movement amplitude and designated target width, respectively. While the executed and imagined movement times are rarely the same due to the differences in base movement times (as indicated by the intercept), they nevertheless demonstrate a close similarity in their relation with task difficulty (as indicated by the slope coefficient) (e.g., Wong et al. 2013).

While this line of research has greatly advocated the equivalence between execution and imagery, it can be argued that it is restricted to findings within the temporal domain. That is, it pertains to situations where only the executed or imagined movement times are evaluated in the presence of particular task constraints. However, there has been comparatively limited research surrounding the equivalence between execution and imagery in terms of the spatial domain. At the same time, it is also possible to reflect the fore mentioned trade-off between movement speed and accuracy by inversely examining the end locations when movement times are held constant (Carlton 1994; Schmidt et al. 1979; Wright and Meyer 1983; Zelaznik et al. 1981). Thus, it is possible to ask whether motor imagery comprises of the same spatial characteristics as physically executed movements once constraints are placed on the movement times.

With this in mind, it is noteworthy that increasing movement velocity (synonymous with a decreased movement time at set amplitude) can increase the within-participant variability of the end location (Schmidt et al. 1979). This finding highlights how the noise or stochastic properties of human movement are signal-dependent–greater impulses manifest in more noise (see also, Faisal et al. 2008; Meyer et al. 1988). This approach to the trade-off between movement speed and accuracy can be formally expressed by a linear function that is sometimes referred to as Schmidt’s Law. This law of human movement may be calculated as: *W*_*e*_ = *a* + *b*(*D*/MT), where *a* and *b* once more represent the intercept and slope coefficient, respectively. Meanwhile, the *D* and *MT* represent the distance and movement time (equating to velocity), and the *W*_*e*_ represents the effective target width, which may be calculated from the within-participant standard deviation (SD) of end locations: SD × 4.133. This spatial parameter is based on the assumption that the end locations across a series of movement attempts reflect a normal or Gaussian distribution, which may be scaled to a hypothetical target boundary that subtends a select proportion of the end locations. In this regard, a multiple of 4.133 represents approximately 95% of the distribution (Welford 1968).

To this end, the aim of the present study was to examine whether motor imagery similarly reflects the signal-dependent noise that typically manifests within execution. To investigate, we had participants execute or imagine a single, horizontal aiming movement with the upper-limb at a pre-specified criterion time. The imagined movements unfolded by having the participants vertically lift and lower the limb to index the start and end of their movements, respectively. In addition, participants precisely estimated, where they imagined their movements to have ended relative to the intended target once the imagery was completed.

Consequently, there were two measures of imagined location: within-trial; when the limb was not intended to deviate from its original location, and post-trial; when the limb was recalled from its previous imagined location. These measures recognise a distinction that is often drawn by neuroscientific and behavioural models (e.g., Csibra 2007; Henke 2010; Shanks 2010; Shriffin and Schneider 1977). Indeed, the former measure is indicative of covert localisation, where the utility of a common neural representation for execution and imagery may unintentionally contaminate the end location (i.e., subtle variations in the end location relative to the start location) (for examples, see Dijkerman and Smit 2007; Kilner et al. 2003; Ray et al. 2013; Roberts et al. 2015). This measure is synonymous with implicit or procedural forms of memory, and it is highly sensitive to lower-level or bottom-up processes. Alternatively, the latter measure indicates overt localisation, where the imagined outcome of movements may be successfully recalled after the imagery has been completed. This measure more greatly captures explicit or declarative forms of memory, and pertains to higher-level or top-down processes. By featuring both of these measures, it is possible to corroborate each of their outcomes, while avoiding any failure in detecting a possible influence within imagery.

To this end, the feature of signal-dependent noise within the human sensorimotor system has been closely reflected by the findings of the W_e_ being positively related to the movement velocity (or inversely related to the movement times at set amplitude) (Schmidt et al. 1979). In other words, the spatial variability is contingent upon the magnitude of the efferent motor signals that are directly responsible for the physical execution of target-directed movements. Thus, it stands to reason that the *W*_*e*_ will be inversely related to the criterion movement times within execution. However, owing to the limited magnitude or complete absence of efferent signals during motor imagery, it is predicted that there will no such relation within imagery. Specifically, there will be an increase in the W_e_ following decreases in movement time for execution, although there will be limited differences in the W_e_ across movement times for imagery (covert and overt measures).

## Methods

### Participants

An apriori power analysis was initially conducted using G*Power software (version 3.1.9.4; see Faul et al. 2007) including the input parameters of: *α* = 0.05, 1 − *β* = 0.90, and *f* = 0.40 (large). The required effect size was directly adapted from a collection of studies that featured statistical main effects of temporal- and accuracy-constraints for measures of effective target width (e.g., Slifkin and Eder, 2017) and movement time (e.g., Heremans et al. 2011; McCormick et al. 2013), respectively. Likewise, it can be indirectly supported by the strong correlation coefficients that are evidenced between the effective target width and movement velocity (Schmidt et al. 1979), as well as the movement time and index of difficulty (Fitts 1954; Fitts and Petersen 1964) (*rs* > 0.90). There was a minimum requirement of 15 participants for this particular study. There were 17 participants (age range = 21–40; 14 male and 3 female; 15 right-handed and 2 left-handed (self-declared)) with normal or corrected-to-normal vision and free from any neurological condition that agreed to take part in the study. The study was designed and conducted in accordance with the Declaration of Helsinki (2013), and approved by the local research ethics committee.

### Materials

**Fig. 1:**
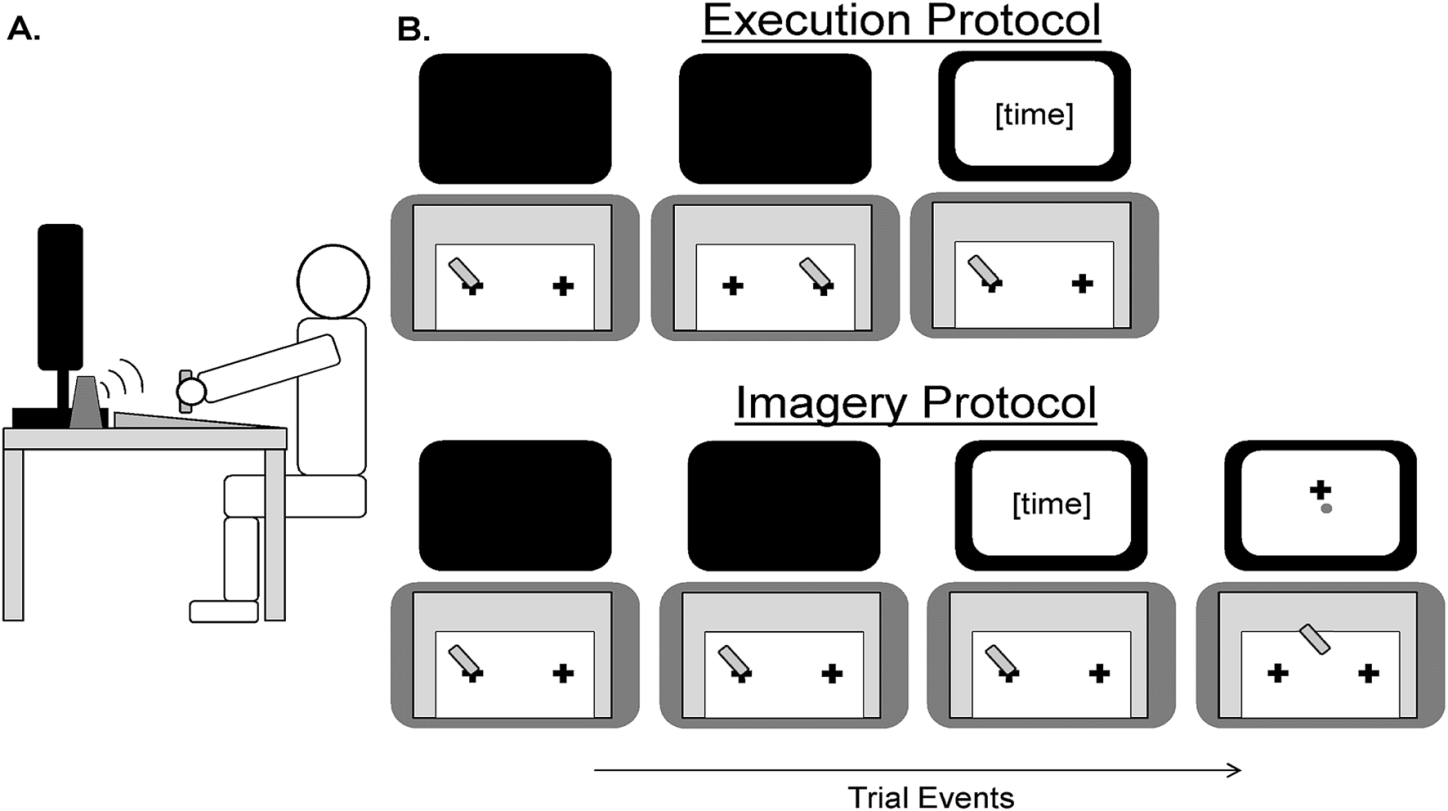
Representative illustration of the experimental set-up (**a**), and trial proceedings for the execution and imagery protocols (**b**). Notably, the imagery protocol features an additional phase for spatial estimation

Participants sat directly in front of a digitizing graphics tablet (GTCO Calcomp Drawing Board VI; temporal resolution = 125 Hz, spatial resolution = 1000 lines per inch) and LCD computer monitor (47.5 cm × 27.0 cm; temporal resolution = 75 Hz; spatial resolution = 1280 × 800 pixels) (Fig. 1a). Targets were represented as black cross-hairs (*L* × *W* = 20 × 2 mm) that were printed on white A4 paper with a 24-cm separation (centre-to-centre), which was secured to the active surface of the tablet by placing it under an attached translucent polypropylene sheet. Stimuli that were generated on the monitor were controlled by Matlab (2018b) (The Mathworks Inc., Natick, MA) running Psychtoolbox (version 3.0.11) (Pelli 1997).

### Execution protocol

Participants were tasked with executing a single, three-dimensional aiming movement in the horizontal medio-lateral direction (right-to-left for right-handed participants, left-to-right for left-handed participants) toward one of the cross-hairs using a stylus pen on the tablet with their dominant upper-limb (Fig. 1b). A trial would commence by initially contacting the surface of the tablet with the tip of the stylus pen. After a 2-s delay, an auditory tone would sound to indicate the start of the trial. Participants were then free to commence the movement in their own time. The start and end of the movement was taken as participants initially lifting the stylus from the home position and once more establishing contact with the tablet surface near the target position, respectively.

Participants had to reach as close as possible to the centre of the target at a pre-specified criterion time. Movement time was allowed to vary ± 10% of the criterion time to qualify as a successful trial. There were three possible criterion times including 400 ms (± 40), 600 ms (± 60) and 800 ms (± 80). Movement time was calculated as the difference in time between the initial lift and subsequent contact with the tablet. Terminal feedback of the movement time was immediately displayed on the monitor following the completion of each movement. If the feedback appeared in green, then it would indicate that the participants successfully reached the criterion time and could move onto the next trial. If the feedback appeared in red, then it would indicate that the participants failed to reach the criterion time and needed to repeat the trial. Participants had to a press key on the adjacent keypad that was connected to the computer to remove the feedback and move on to the next attempt.

### Imagery protocol

Participants were tasked with imagining the same aiming movement as the execution protocol (Fig. 1b). Likewise, the trial events unfolded in a similar fashion to the execution protocol, although the start and end of the movement was indexed by participants initially lifting the stylus before once more establishing contact with the tablet surface near the home position.

Participants had to start and end their imagined movements at the same pre-specified criterion times as the execution protocol. Because the physical movements during imagery accumulated minimal displacement compared to execution, we sought to corroborate the potentially subtle variations in the end location within trials. Thus, if the imagined movement successfully reached the criterion time, then the participants were prompted to additionally estimate the imagined location of their limb following the completion of imagery. Specifically, participants had to move the stylus on the tablet to direct a cursor around the area of the cross-hair that was displayed on the monitor. When the participants perceived themselves to be positioned at the same location as the end of the imagined movement relative to the target, then they simply held the position of stylus while selecting an adjacent button near the tip of the stylus. If the imagined movement failed to reach the criterion time, then participants were not prompted to estimate their imagined location and needed to repeat the trial.

### Block procedures

As well as being familiarised with the aiming task, it was essential for participants to be initially familiarised with the criterion times prior to formally undertaking the execution and imagery protocols. Likewise, the equivalence between execution and imagery is often contingent upon the prior practice or physical exposure to the task dynamics, where a representation may be initially constructed in practice and later awakened in imagery (Yoxon et al. 2015, 2017). Thus, participants undertook one set of practice trials when they were first introduced to each of the criterion times. The practice trials were completed immediately prior to one of the execution or imagery protocols depending on their order. The practice trials were similar to the execution protocol including the provision of terminal feedback of the movement time. In the event that participants failed to reach a criterion time, then they had to repeat the trial. Practice continued to unfold until the participants reached the criterion time on ten trials.

The criterion times were undertaken in a blocked order of trials, which were counter-balanced between participants using a Latin-square design. Within each block of criterion times, participants completed the execution and imagery protocols in a blocked order of trials. The order of the execution and imagery protocols within each block of criterion times was also counter-balanced between participants. The execution and imagery protocols would continue to unfold until the participants reached the criterion time on 15 trials before subsequently progressing on to the next criterion time.

### Data management

Position data from the tablet were stored as pixelated coordinates from the monitor. Positional errors were calculated within the horizontal (*x*-axis) and vertical (*y*-axis) directions and converted into millimetres. For the executed and imagined movements, positional error was regarded as the distance between the end location and target, which was taken as the participant mean start location plus the movement amplitude (24 cm). For the estimated imagined location, positional error was regarded as the distance between the estimated location and centre of the cross-hair location on the monitor.

While the aiming task was an unconstrained three-dimensional movement that generates measures within the horizontal and vertical directions, the theoretical underpinnings and empirical research principally relate to the central tendency and dispersion of the primary axis alone (e.g., Elliott et al. 2017; Meyer et al. 1988; Schmidt et al. 1979; Slifkin and Eder 2017; Zelaznik et al. 1981; cf. Carlton 1994). Thus, the dependent measures were derived from the primary direction of the movement (i.e., horizontal; *x*-axis). The signed difference in the horizontal position of the limb and target for each of the individual trials was initially calculated. Therein, the within-participant standard deviation (SD) (population *n* degrees-of-freedom) was calculated, and converted by a multiplicative value of 4.133 to obtain the W_e_: SD × 4.133 (Welford 1968). Prior to these calculations and formal statistical analysis, trials were removed when participants mistakenly failed to move during the execution protocol (< 15-cm movement amplitude), definitively moved during the imagery protocol (> 15-cm movement amplitude) or failed to reach the criterion movement times (± 10%).^1^

In addition to the W_e_ itself, we also calculated the individual participant slope coefficients that pertained to the relation between W_e_ and MT. While the W_e_ is linearly related to velocity (i.e., *W*_*e*_ = *a* + *b*(*D*/MT)), it is not possible to derive this parameter from imagined movements. Nevertheless, a linear relation between W_e_ and velocity should translate into a similar relation between W_e_ and MT when *D* is held constant (24 cm).

### Statistical analysis

In line with the principles of open science, the individual participant data and calculations for the relevant measures have been uploaded to the Open Science Framework: https://osf.io/d3n6u. We separately analysed the actual imagined locations within the trials and estimated imagined locations after the trials to indicate both covert (synonymous with subtle unintended end locations) and overt (synonymous with consciously selected end locations) aspects of spatial localisation, respectively. Covert localisation of the limb was first assessed by analysing the W_e_ using a two-way repeated-measures ANOVA, which featured the factors of protocol (execution, imagery) and temporal window (400 ms, 600 ms, 800 ms). Depending on an assessment of normal distribution using the Shapiro–Wilk test, we compared the participant slope coefficients of execution and imagery using a paired sample t-test (parametric) or Wilcoxon signed-rank test (non-parametric). Meanwhile, the overt localisation of estimated imagined locations was assessed by analysing the W_e_ across the criterion times using a one-way repeated-measures ANOVA. Providing confirmation of the data being normally distributed, the participant slope coefficients were compared to a theoretical value of zero using a single-sample t-test (synonymous with no linear relation).

Significant interactions from the factorial ANOVA were initially decomposed by conducting simple effect analyses on each level of protocol (using the mean square error and degrees-of-freedom of error from the original factorial ANOVA). Further significant effects that featured more than two means were decomposed using the Tukey HSD post hoc procedure. Effect sizes from the ANOVAs were indicated by partial eta-squared (*η*_*p*_^*2*^). All statistical effects were declared as significant at *p* < 0.05.

## Results

### Covert localisation

**Fig. 2:**
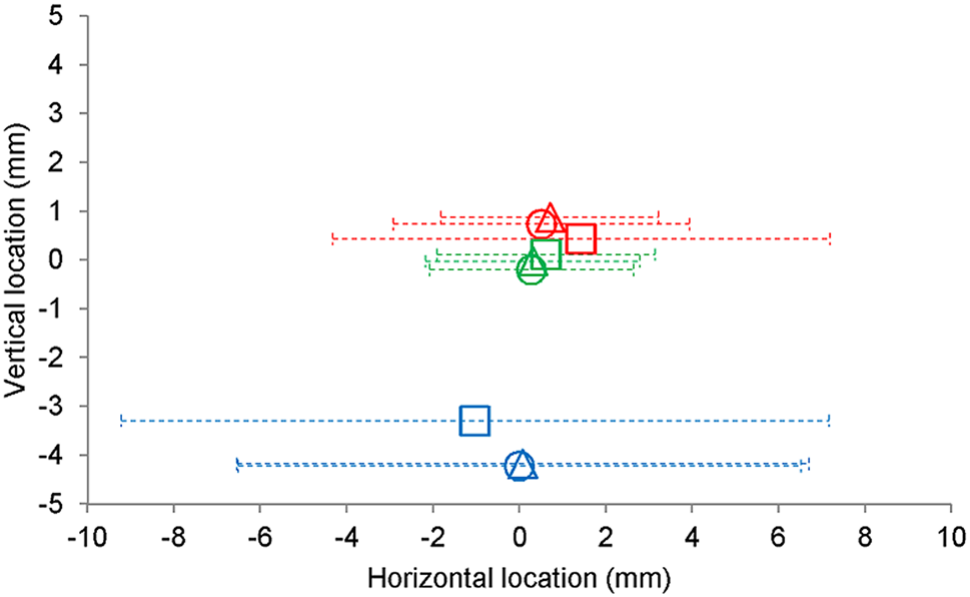
Illustration of the mean horizontal and vertical locations with respect to the target (0, 0) for execution (red) and imagery (covert = green, overt = blue). Symbols indicate the different temporal windows (400 ms = squares; 600 ms = circles; 800 ms = triangles). Dotted error bars collectively span the *W*_*e*_ (equivalent to 95% of the distribution)

**Fig. 3:**
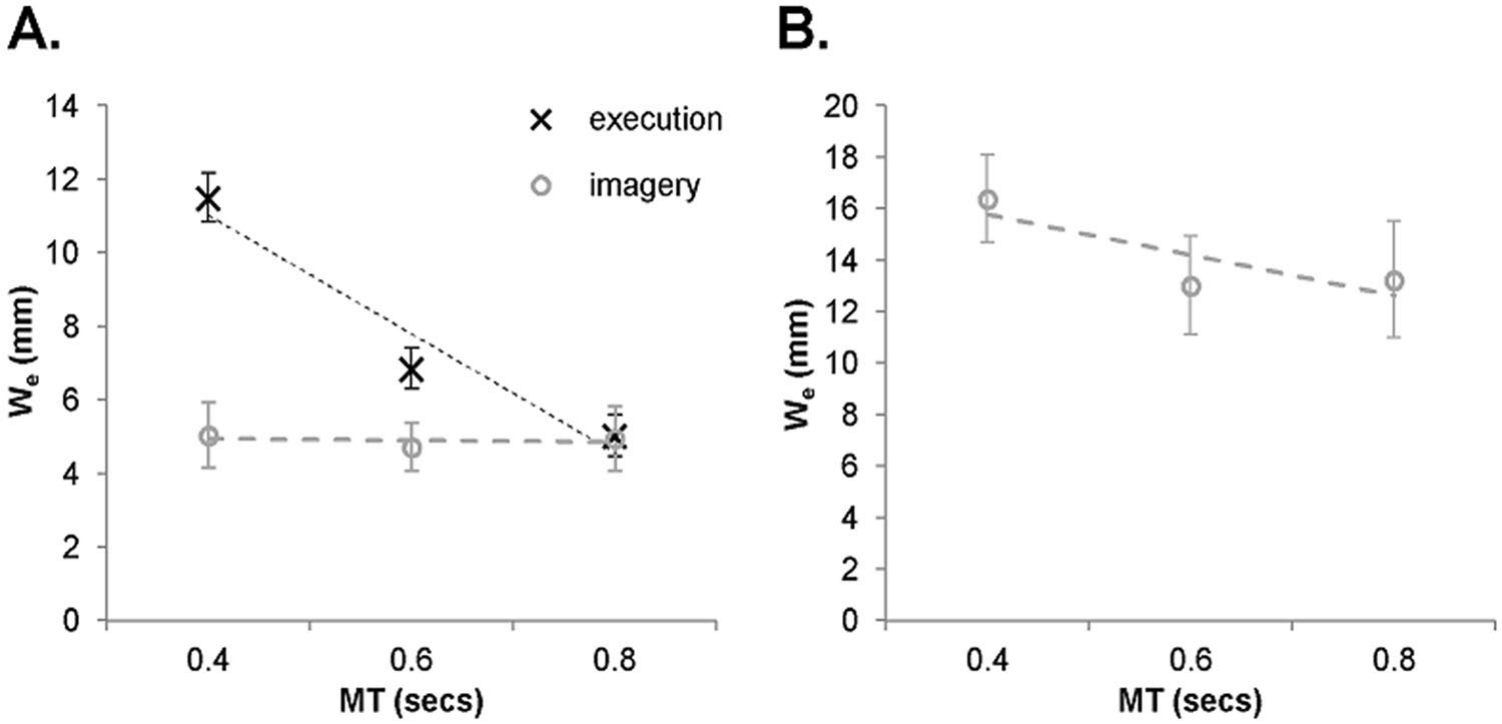
Mean W_e_ as a function of temporal window for execution and imagery (covert) (**a**), and spatial estimates following imagery (overt) (**b**). Error bars indicate between-participant standard error of the mean

Analysis of the W_e_ indicated a significant main effect of protocol, *F*(1, 16) = 27.30, *p* < 0.001, *η*_*p*_^*2*^ = 0.63, and temporal window, *F*(2, 32) = 16.44, *p* < 0.001, *η*_*p*_^*2*^ = 0.51. These statistical effects were superseded by a significant interaction between protocol and temporal window, *F*(2, 32) = 26.59, *p* < 0.001, *η*_*p*_^*2*^ = 0.62 (Figs. 2, 3a). Simple effect analyses on each level of protocol revealed a significant main effect of temporal window for execution, *F*(2, 32) = 55.89, *p* < 0.001, *η*_*p*_^*2*^ = 0.78. Post hoc analysis revealed a significantly larger width for the 400-ms window compared to the 600-ms window, which was also significantly larger than the 800-ms window (Tukey HSD value = 1.56).^2^ However, there was no significant main effect of temporal window for imagery, *F*(2, 32) = 0.14, *p* > 0.05, *η*_*p*_^*2*^ = 0.00. Comparison of the individual participant slopes that pertain to the W_e_-MT relations indicated a significantly more negative slope for execution (Mdn = − 14.89, IQR = 13.81) compared to imagery (Mdn = 0.17, IQR = 6.34), *T* = 1, z = − 3.57, *p* < 0.001.

### Overt localisation

Analysis of the W_e_ indicated that there was no significant main effect of temporal window, *F*(2, 32) = 0.1.14, *p* > 0.05, *η*_*p*_^*2*^ = 0.07 (Figs. 2, 3b). Meanwhile, the individual participant slopes indicated no significant difference compared to a theoretical value of zero (*M* = − 8.46, *SE* = 6.37), *t*(16) = 1.33, *p* > 0.05.

Following observation of the mean and within-participant variability (Fig. 2), we observed a much larger degree of error from the estimates of imagined end locations compared to the covert measures. To explore this trend further, we additionally compared the total error of executed and estimated imagined locations (synonymous with the within-participant root mean square error) within the primary and secondary direction of movement. For the primary direction, there was significantly less error produced by execution (*M* = 2.47, SE = 0.15) compared to the estimates following imagery (*M* = 4.41, SE = 0.40), *t*(16) = 4.34, *p* < 0.01. Likewise, for the secondary direction, there was significantly less error for execution (Mdn = 1.91, IQR = 0.40) compared to the estimates following imagery (Mdn = 4.68, IQR = 16.27), *T* = 6, *z* = − 3.34, *p* < 0.01. Because the current executed and estimated imagined locations were captured by separate measures (i.e., within-trial vs. post-trial), where any differences between them could merely result from some other independent process (e.g., memory), these findings should be interpreted with at least some degree of caution.

## Discussion

The present study examined whether the increasing spatial variability that is exhibited following decreases in executed movement times may also be reflected in motor imagery. That is, we asked whether the inverse relation between W_e_ and criterion movement time for executed movements may correspondingly manifest in the covert or overt localisation of imagined movements. Both these measures ensured that non-conscious lower-level and conscious higher-level components of the imagined movement locations could be evaluated, respectively. Broadly speaking, the findings showed that the W_e_ was inversely related to the criterion movement time only for execution, and not for imagery. At the same time, there was an increased error in the post-trial estimates of the imagined end locations compared to the actual executed movements.

The inverse relation between the W_e_ and movement time pertains to the inherent trade-off between movement speed and accuracy, where the noise or stochastic properties of human movement are signal-dependent (Faisal et al. 2008; Meyer et al. 1988; Schmidt et al. 1979). In other words, these random sources of spatial variability are contingent upon the projection of efferent motor signals. Thus, it stands to reason that with the comparatively limited efferent motor signals during imagery, there is limited semblance to the downstream consequences on covert spatial localisation. That said, the limited equivalence between the spatial characteristics of execution and imagery within the present study alludes to mere random sources of variability as opposed to intended or pre-planned sources of variability (van Beers 2009). Indeed, it is still possible that the neural representation underlying the equivalence between execution and imagery may extend to the spatial characteristics of movement, but only when it originates from intended sources (i.e., pre-planned direction of movement). For example, in a similar vein to the execution of bimanual movements, there is evidence that the executed straight-line movements of one limb can become contaminated and begin to coordinate with the imagined circular movements of the unused limb (Piedimonte et al. 2018; see also, Ramsey et al. 2010). In this instance, the imagined movement direction may have awakened a neural representation that is correspondingly designed for the physical execution of that same movement direction (Jeannerod 1994, 1999).

Because of the limited similarity in the trends that emerge between the movement times within executed and imagined movements, it is debatable whether the current measures of covert and overt localisation even capture the correspondence between execution and imagery. Indeed, while there were limited differences in the within-participant variability of imagined movements, it is possible to evidence a correspondence between execution and imagery by more closely observing the between-participant variability. That is, the between-participant variability that often manifests in executed movements may also unfold within the imagined movements (for a similar logic, see Welsh et al. 2009; Welsh and McDougall 2012). With this in mind, further correlations (Spearman’s rho; non-parametric) on the W_e_ indicated a positive relation between the executed and imagined movements (covert 400 ms: *r*_*s*_ = 0.36, *p* > 0.05; 600 ms: *r*_*s*_ = 0.67, *p* < 0.001; 800 ms: *r*_*s*_ = 0.56, *p* < 0.05; overt 400 ms: *r*_*s*_ = 0.19, *p* > 0.05; 600 ms: *r*_*s*_ = 0.05, *p* > 0.05; 800 ms: *r*_*s*_ = 0.38, *p* > 0.05). This relation may be interpreted as the characteristics of executed movements spilling-over onto imagined movements, where the more precise individuals are in execution, then the more precise they are in imagery. Presumably, the representation that is responsible for appropriately parameterizing executed movements is the same representation that is utilised for imagined movements. However, the relation between executed and imagined movements was only evident within the covert measure (as opposed to overt localisation), which would suggest that these effects pertained to a lower-level, downstream consequence of utilising a neural representation that appears outside of conscious awareness (for similar covert responses, see Kilner et al. 2003).

Despite the limited similarity between the executed and overt measure of imagined movements, the additional analysis of total error indicated a modest and more erred spatial estimate for imagery compared to the actual error for execution. This finding may refute potential concerns surrounding participant engagement within the imagery protocol as this possibility would have alternatively manifested in a smaller amount of error (i.e., near perfection). Instead, it is possible that participants were consciously aware of the spatial variability that could manifest from executed movements (constant error (CE) grand* M* = 0.87 mm, *W*_*e*_ grand* M* = 7.80 mm; hypothetical range of error (CE ± *W*_*e*_/2) = − 3.03–4.77 mm), and consequently estimated over a larger area that could safely fit the distribution of actual end locations (CE grand* M* = -0.32, *W*_*e*_ grand* M* = 14.22 mm; hypothetical range of error (CE ± *W*_*e*_/2) = − 7.43–6.79 mm). This trend concurs with computational models of sensorimotor control, where the nervous system converges onto a movement approach or central tendency that compensates for the distribution and associated likelihood of movement outcomes (Harris and Wolpert 1998; Wolpert and Ghahramani 2000). For example, it has been shown that individuals typically undershoot intended target locations to avoid the cost of overshooting when they initially perceive an increased likelihood of missing the target (Elliott et al. 2004; Roberts 2020).

Alternatively, it is possible that the overly erred estimate of imagined end locations following the pre-specified criterion times may resemble the previously evidenced increase in imagined compared to executed movement times under particular task constraints. That is, the base movement times (as indicated by the intercept from the Fitts’ Law equation) tend to be greater for imagined compared to executed movements when the task difficulty is altered and time is no longer constrained (e.g., Wong et al. 2013). This trend has been primarily attributed to the additional demands placed on the control of imagined movements, including the need to index the start and end of the movements (e.g., lifting the stylus before returning to a similar position) (Glover and Baran 2017; Glover and Dixon 2013; for an alternative explanation, see Yoxon et al. 2015, 2017). In the context of the present study, it is possible that the additional demands that are placed on the control of imagined movements could no longer be accommodated within the movement time, because it was constrained, and thus alternatively manifested in a larger estimate of spatial error.

At the same time, it is also important to recognise the underlying contributions of planning and control within executed movements to appreciate the overly erred estimate of imagined end locations. Indeed, the early portion of executed movements (e.g., prior to peak velocity) are usually subject to a degree of error, where the parameterization of movement prior to the movement itself is not adequate enough to reach the intended target (van Beers 2009; for measuring the contribution of planning/control, see Elliott et al. 1999; Heath et al. 2004). Consequently, it is possible to utilise sensory feedback within the movement (e.g., after peak velocity) to adjust the trajectory whenever an error ensues (Elliott et al. 2001). In the context of imagery, the utility of a representation that is correspondingly used for the initial execution of movement may solely contribute to the outcome of imagined movements because of the absence of any movement-specific sensory feedback. Thus, while it is possible for imagery to infer and perhaps compensate for the error that is associated with planning, it is unable to overcome this error in a way that can be achieved during execution (for a similar logic, see Glover and Baran 2017; Glover and Dixon 2013).

In conclusion, while it is well accepted that motor imagery is consistent with the trade-off between movement speed and accuracy when using the Fitts aiming paradigm (temporal domain), the present study shows how it is not entirely consistent with the trade-off when alternatively adopting the Schmidt aiming paradigm (spatial domain). That is, imagined movements fail to account for the signal-dependent noise that typically manifests within executed movements. This trend was demonstrated in both covert and overt measures of spatial localisation. Thus, we contend that while motor imagery encompasses a neural representation that is correspondingly used for execution, it is limited to intentional or pre-planned components of movement without accurately incorporating the noise or stochastic properties of the movement itself. Future research may similarly explore whether motor imagery encapsulates the spatial characteristics of executed movements, including the separate contribution of intended and random sources of spatial variability.

## Footnotes

1 The number of attempts where participants failed to reach each of the criterion times (± 10%) were analysed using a two-way repeated-measures ANOVA, which featured factors of protocol (execution, imagery) and temporal window (400 ms, 600 ms, 800 ms). There was a significant main effect of protocol, *F*(1, 16) = 56.11, *p* < .001, *η*_*p*_^*2*^ = 78, as there were significantly fewer trials with a temporal error for execution (*M* = 12 trials, SE = 2.00) compared to imagery (*M* = 23 trials, *SE* = 1.14). There was a significant main effect of temporal window, *F*(2, 32) = 104.02, *p* < 0.05, *η*_*p*_^*2*^ = 0.22. Post hoc analysis revealed that there were significantly more errors generated for the 400- (*M* = 21 trials, SE = 2.44) compared to 800-ms window (*M* = 14 trials, SE = 1.84), while there were no significant differences for the comparisons to the 600-ms window (*M* = 18 trials, SE = 1.77) (Tukey HSD value = 6.10). Meanwhile, there was no significant interaction between protocol and temporal window, *F*(2, 32) = 0.02, *p* > 0.05, *η*_*p*_^*2*^ = 0.01.

2 Trend analyses for the execution protocol indicated both a significant linear, *F*(1, 16) = 45.76, *p* < 0.001, *η*_*p*_^*2*^ = 0.74, and quadratic, *F*(1, 16) = 7.74, *p* < 0.05, *η*_*p*_^*2*^ = 0.33, component. While reflecting a negative linear relation between W_e_ and MT, there appeared less of a decrease between the 600- and 800-ms windows. This outcome is not without precedence as previous findings have shown a reduced linear relation for longer criterion movement times (e.g., Schmidt et al. 1979). Presumably, the more prolonged temporal windows (≥600 ms) accommodate a feedback processing time-lag, where online adjustments to the trajectory may serve to reduce the spatial variability (Zelaznik et al. 1981; see also, Elliott et al. 2001).
